# A stability indicating HPLC method for the determination of clobazam and its basic degradation product characterization

**DOI:** 10.1186/2008-2231-22-49

**Published:** 2014-06-11

**Authors:** Effat Souri, Amin Dastjani Farahani, Reza Ahmadkhaniha, Mohsen Amini

**Affiliations:** 1 Department of Medicinal Chemistry, Faculty of Pharmacy and Drug Design and Development Research Center, Tehran University of Medical Sciences, Tehran 14155-6451, Iran; 2 Department of Human Ecology, School of Public Health, Tehran University of Medical Sciences, Tehran 1417614411, Iran

**Keywords:** Clobazam, HPLC, Stability indicating, Stress degradation

## Abstract

**Background:**

Clobazam is used for the treatment of different types of seizure and epilepsy. The present research is undertaken to study the systematic forced degradation of clobazam and to identify its main degradation product under basic conditions.

**Methods:**

The degradation of clobazam was studied under different conditions. Clobazam and its degradation products were separated using a Nova-Pak C18 column and a mixture of KH_2_PO_4_ 50 mM (pH 8.5) and acetonitrile (50:50, v/v) as the mobile phase with UV detection at 230 nm.

**Results:**

The within-day and between-day precision values in the calibration range of 0.1-20 μg/ml were within 0.5-1.5%. Clobazam was relatively stable in solid from under exposure to visible and UV light and also heat. The clobazam aqueous solution of clobazam was more labile under exposure to visible and UV light. The bulk drug was significantly degraded under exposure to 2 M HCl, 0.1 M NaOH or 3% H_2_O_2_. Using the tablet powder, higher degradation rates were observed under different stress conditions. The main degradation product of clobazam under basic condition was subsequently characterized.

**Conclusion:**

The developed method could be used for the determination of clobazam in the presence of its degradation products with acceptable precision and accuracy. The applicability of the proposed method was evaluated in commercial dosage forms analysis.

## Introduction

**Figure 1:**
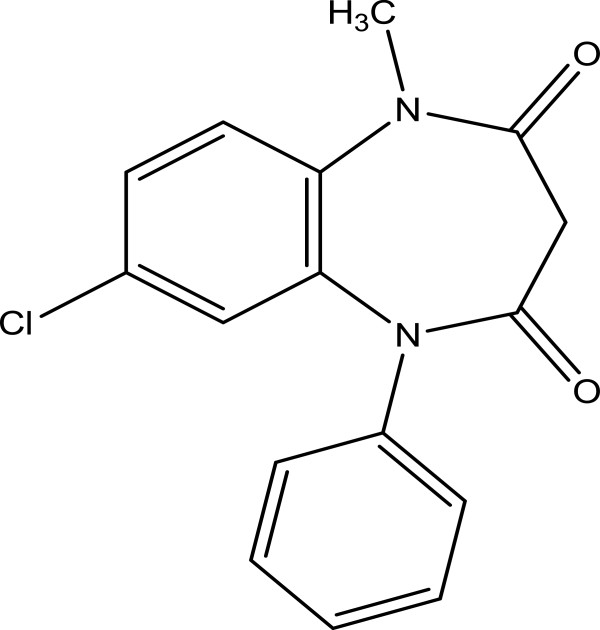
Chemical structure of clobazam.

Clobazam, 7-chloro-1-methyl-5-phenyl-1, 5-benzodiazepine-2, 4 (3H-dione) (Figure 1), is a benzodiazepine derivative which is used for the treatment of various seizure types and epilepsy [1]. Clobazam belongs to the 1, 5-benzodiazepine class with a pk_a_ value of 6.65. Clobazam bulk powder is a white crystal with molecular weight of 300.7. The drug is slightly soluble in water and soluble in alcoholic solvents. A number of HPLC methods have been reported before for the determination of clobazam and its metabolite in human plasma [2-6]. Also, HPLC method has been developed for the determination of clobazam in tablet dosage forms [7]. Clobazam is official in British Pharmacopeia (BP) and The United States Pharmacopeia (USP) and an HPLC method is reported for the assay determination of this drug in capsule dosage forms.

Literature survey showed that there is no stability indicating HPLC method for the determination of clobazam in the presence of its degradation products or its degradation behavior under stress conditions. Hence, it is important to develop an accurate, rapid and specific stability indicating analytical method, which is suitable for routine quality control analysis of clobazam in pharmaceutical dosage forms. Stress testing provides important information about the stability of the drug substance under different conditions. Moreover, the suitability of the proposed analysis technique could be verified. In this study, reversed-phase HPLC method is proposed for the determination of clobazam in bulk drug and pharmaceutical dosage forms. The stability tests were performed under different conditions according to the International Conference on Harmonization (ICH) guidelines to find out the suitability of the proposed analytical technique in the presence of degradation products.

## Methods

### Chemicals

The clobazam bulk powder was kindly provided by Hakim Pharmaceutical Company (Tehran, Iran). HPLC grade acetonitrile and analytical grade potassium dihydrogen phosphate were purchased from Merck (Darmastadt, Germany). Clobazam tablets (10 mg) were from Dr Abidi Pharmaceutical Laboratory, Tehran, Iran and obtained from a local pharmacy.

### Instrumentation

The analysis was performed by using a chromatographic system from Waters (Milford, USA) consisting of a Model 515 isocratic pump, a Model 710 plus autosampler and a Model 480 UV–vis detector. The data processing system was the version 1.5x of a multi-channel Chrom&Spec software for chromatography. For heat studies, a dry air oven (Melag, Germany) was used. A 100 W Tungsten lamp and a low-pressure Mercury lamp 100 W were used as visible or UV light source, respectively.

The ^1^H-NMR spectrum was obtained in CDCl_3_ using a Bruker FT-500 Spectrometer (Bruker, Rheinstetten, Germany). The chemical shifts (δ) relative to tetramethylsilane as internal standard were reported in part per million (ppm). The mass spectrum was achieved on an Agilent 5975C Spectrometer. The IR spectrum was obtained by using a Nicolet 550-FT Spectrophotometer (Nicolet, Maison, WI, USA).

Peak purity of the samples resulted from stress degradation was checked using an Agilent 1100 HPLC system (Agilent Technologies, USA), equipped with a quaternary pump, an on-line degasser, an auto-sampler, a column oven, and a Photodiode Array (PDA) detector coupled with a ChemStation Software version B.04.01 (Agilent Technologies, USA). For the peak purity analysis, the spectra were corrected for background absorption caused by the mobile phase or matrix compounds, by subtracting the appropriate reference spectra. The spectra were assessed in the range of 225–254 nm and the mode of “best possible match of the entire spectrum” was selected as the mode of normalization.

### Chromatographic conditions

A Nova-pak C_18_ column (150 mm × 3.9 mm, 4 μm, Waters, Milford, USA) was used for the chromatographic separation. The mobile phase was composed of 50 mM KH_2_PO_4_ (pH 8.5) and acetonitrile in the ratio of 50:50 (v/v) and pumped at room temperature, at a flow rate of 1 ml/min. The wavelength of the UV detector was set at 230 nm. The mobile phase was degassed by filtration through a 0.45 μm filter and sonication for 5 min.

### Stock standard solutions

A 50 μg/ml stock standard solution of clobazam was prepared in acetonitrile. Calibration solutions of clobazam at 0.1, 0.2, 0.5, 1, 2, 5, 10 and 20 μg/ml were prepared by transferring various aliquots of clobazam solution (50 μg/ml) into a series of 10 ml volumetric flasks and completing the volume to the mark with the mobile phase.

### Linearity

Six sets of calibration standard solutions of clobazam were prepared in mobile phase in the range of 0.1-20 μg/ml. The solutions were analyzed according to the optimized chromatographic conditions and the peak area was plotted over the clobazam concentration. The statistical data were calculated.

### Precision and accuracy

Three different concentrations of clobazam (0.2, 2 and 20 μg/ml) within the calibration range were prepared and analyzed in triplicate during one day and three consecutive days. The coefficient of variation (CV) and error values were determined to find out the precision and accuracy of the analytical method.

### Robustness

To find out the robustness of the proposed method, the chromatographic conditions were changed. The influence of the pH value of the mobile phase and also the percent composition of the mobile phase were studied.

### Analysis of tablets

The average weight of twenty finely powdered clobazam tablets (10 mg) was calculated. An amount of powdered tablet equivalent to one tablet was weighed and transferred into a 100 ml volumetric flask. After addition of about 70 ml of water and acetonitrile (50:50, v/v), the mixture was vortex-mixed for 15 min. The solution was diluted to mark with the same solvent and centrifuged at 4000 rpm for 10 min. The solution was subjected to the analysis method after four times dilution and filtration through a 0.45 μm polypropylene syringe filter (Teknokroma, Spain).

### Recovery

The standard addition method was used to evaluate the relative recovery of clobazam from dosage forms. Standard concentrations of clobazam were added to a solution resulted from tablet sample and analyzed. The resulted peak area was compared with a standard solution at the same concentration level and the relative recovery was calculated.

### Stress degradation

#### ***Acid and base induced degradation***

Clobazam solutions containing 500 μg/ml in 1 M HCl, 2 M HCl and 0.1 M NaOH were allowed to stand at room temperature or 60°C. For the HPLC analysis, 0.5 ml of the solution was transferred into a 10 ml volumetric flask and the excess of acid or base were neutralized with NaOH or HCl, respectively. After diluting to the mark with the mobile phase, the solution was injected to the HPLC system. The peak area was compared with freshly prepared samples at the same initial concentration value. The same procedure was performed using tablet powder instead of clobazam bulk powder. All experiments were performed in triplicate.

#### ***Hydrogen peroxide induced degradation***

Clobazam solutions in 3% H_2_O_2_ (500 μg/ml) prepared from bulk drug or tablet powder were kept at room temperature or 60°C. After twenty times dilution with the mobile phase, the resulted solution was injected to the HPLC system and the peak area compared with a standard solution at the same concentration.

#### ***Photolytic degradation***

To study the effect of light on drug substance, 100 mg of the bulk powder and also tablet powder were spread in a watch glass and directly exposed to visible or UV-light. The distance between the light source and the samples was 20 cm. For HPLC analysis, 5 mg of the powder was weighed and dissolved in 10 ml mobile phase and injected to the HPLC system after 20 times dilution. Clobazam solutions in water (500 μg/ml) prepared from bulk powder or tablet powder was also exposed to visible or UV-light. The solution was diluted in mobile phase to give a claimed concentration of 25 μg/ml and 20 μl was injected for HPLC analysis. The percentage of the remained clobazam was calculated using a freshly prepared standard solution at the same concentration.

#### ***Heat degradation***

To find out the thermal stability, clobazam bulk powder and tablet powder were incubated in a dry oven at 80°C for 5 days. Also, aqueous solutions of clobazam bulk powder and tablet powder were incubated in a dry oven at 80°C for 5 days. Solution prepared from these samples were injected to the HPLC system and compared with a standard solution to calculate the percent of degradation.

### Isolation of the basic degradation product of clobazam

The degradation product formed in 0.1 M NaOH after 5 h at 60°C was a white crystal. The crystals were separated and dried in a vacuum desiccator. The purity of this product was proved by injecting a sample solution to the HPLC system. The retention time of the product was about 7.5 min. The structure of this product was elucidated by using its ^1^H-NMR, mass and IR spectra.

## Results and discussion

### Chromatographic conditions

**Figure 2:**
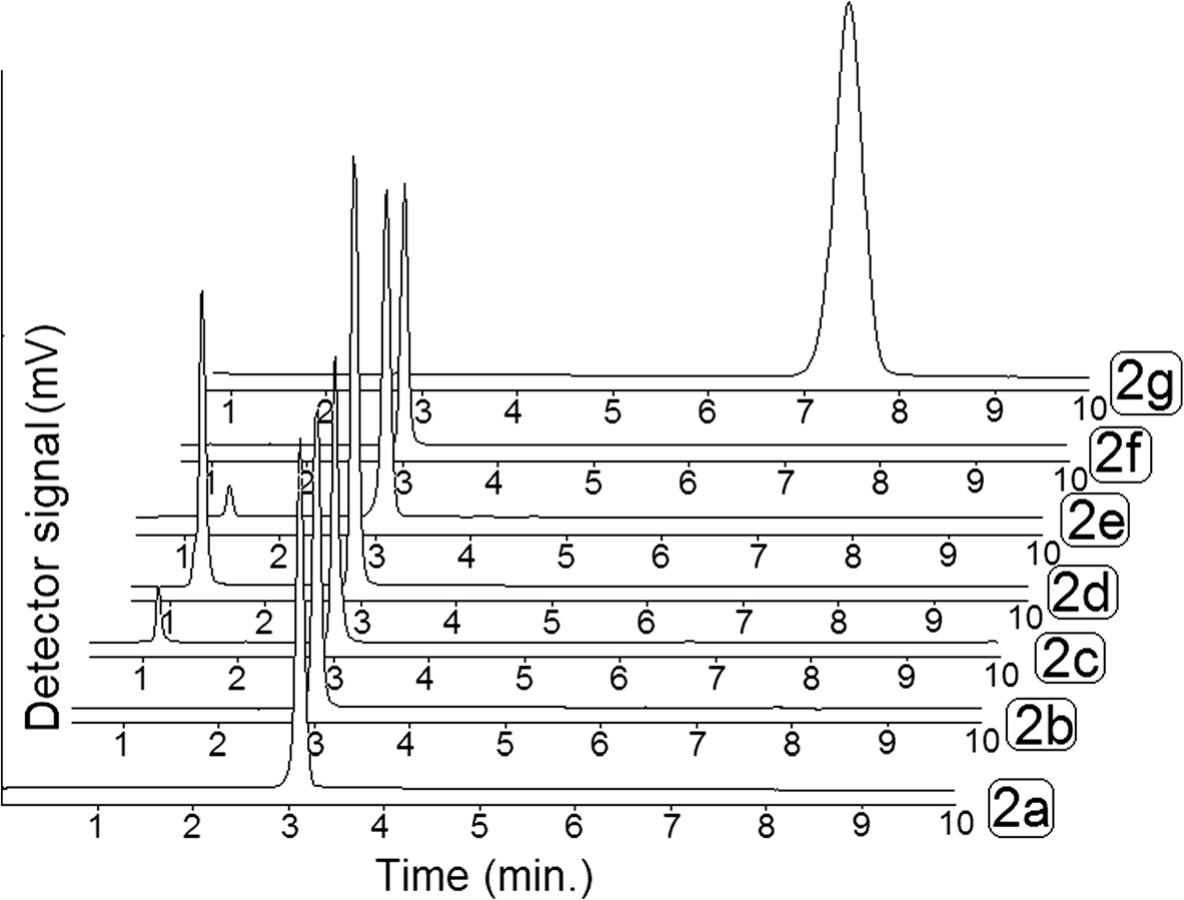
**Typical chromatograms obtained from stability studies of clobazam. (a)** clobazam standard solution (25 μg/ml); **(b)** clobazam solution in 2 M HCl after 1 h at 60°C; **(c)** clobazam solution in 0.1 M NaOH after 1 h at room temperature; **(d)** clobazam solution in 3% H_2_O_2_ after 1 h at 60°C; **(e)** clobazam solution in water after 5 days exposure to UV light; **(f)** clobazam dosage form solution in water after 5 days exposure to heat; **(g)** degradation product of clobazam in 0.1 M NaOH.

By using a Nova-Pak C18 column and a mobile phase consisting of 50 mM KH_2_PO_4_ (pH 8.5) and acetonitrile (50:50, v/v), the clobazam and its degradation products were well resolved. The representative chromatograms (Figure 2) showed no peak interfering from excipients or degradation products with analyte. Analysis of the samples after stress degradation studies showed that the HPLC method is stability indicating.

The system suitability parameters (peak symmetry and repeatability) were evaluated by six replicates injecting of a clobazam solution (25 μg/ml) in mobile phase. The results shown in Table 1 are within the acceptable range.

**Table 1:** System suitability parameters

**Parameters**	**Found**	**Acceptable limits**
USP theoretical plates (n = 6)	4100	N > 1500
USP tailing factor (n = 6)	1.2	T < 1.5
Repeatability (t_R_) (n = 6)	0.48	RSD < 1%
Repeatability (peak area) (n = 6)	0.87	RSD < 1%

*t*_*R*_: Retention time (min); *N*: Theoretical plate; *T*: Tailing factor; *RSD*: Relative Standard Deviation.

### Linearity

Calibration plots over the clobazam concentration range of 0.1-20 μg/ml showed acceptable correlation coefficients. The statistical data of the repeated calibration curves are shown in Table 2. The limit of quantification (LOQ) and limit of determination (LOD) were calculated according to the following equations [8] and are presented in Table 2.

**Table 2:** Statistical data of calibration curves of clobazam (n = 6)

**Parameters**	**Results**
Linearity range	0.1-20 μg/ml
Regression equation	y = 183.39 x + 22.22
Standard deviation of slope	0.89
Relative standard deviation of slope (%)	0.49
Standard deviation of intercept	2.75
Correlation coefficient (r^2^)	0.9997
Limit of quantification (LOQ)	0.15 μg/ml
Limit of detection (LOD)	0.05 μg/ml

LOQ=10σ/sandLOD=3.3σ/s

where σ is the standard deviation of intercept and s is the slope of the calibration graph.

### Stability

The clobazam solutions showed acceptable stability (>99%) relative to freshly prepared standard solutions after 7 days in refrigerator.

### Precision and accuracy

To find out the repeatability of the developed method, triplicate analysis of clobazam solutions at three different concentration levels were performed (in one day and three consecutive days). The calculated CV (%) and errors (%) values are presented in Table 3 which indicates high precision and accuracy of the method.

**Table 3:** Precision and accuracy of the method for determination of clobazam (3 sets for 3 days)

**Concentration added (μg/ml)**	**Concentration found (μg/ml)**	**CV (%)**	**Error (%)**
**Within day (n = 3)**			
0.200	0.199 ± 0.003	1.52	−0.50
2.000	1.977 ± 0.020	1.01	−1.15
20.000	19.967 ± 0.153	0.77	−0.17
**Between day (n = 9)**			
0.200	0.200 ± 0.003	1.51	0.00
2.000	1.980 ± 0.021	1.04	−1.00
20.000	19.940 ± 0.092	0.46	−0.30

Small variations in the pH value of the phosphate buffer and also composition of the mobile phase did not show significant effect on the peak area or peak shape (Table 4) which showed the robustness of the proposed method.

**Table 4:** The influence of small changes in mobile phase composition (method robustness)

**Mobile phase composition**	**Retention time (min)**	**Peak area**
KH_2_PO_4_ 50 mM (pH 8.8)-Acetonitrile (50:50)	3.12	8063
KH_2_PO_4_ 50 mM (pH 8.8)-Acetonitrile (52:48)	3.82	8078
KH_2_PO_4_ 50 mM (pH 8.8)-Acetonitrile (48:52)	3.06	8178
KH_2_PO_4_ 50 mM (pH 8.5)-Acetonitrile (50:50)	3.40	8117
KH_2_PO_4_ 50 mM (pH 8.5)-Acetonitrile (52:48)	3.92	8087
KH_2_PO_4_ 50 mM (pH 8.5)-Acetonitrile (48:52)	3.13	8147
KH_2_PO_4_ 50 mM (pH 8.2)-Acetonitrile (50:50)	3.14	8205
KH_2_PO_4_ 50 mM (pH 8.2)-Acetonitrile (52:48)	3.59	8180
KH_2_PO_4_ 50 mM (pH 8.2)-Acetonitrile (60:52)	3.12	8107

### Recovery

Recovery was evaluated by the determination of the analyte in solutions prepared by the standard addition technique. The mean percentage recovery of 98.3 ± 0.5% was achieved.

### Degradation studies

#### ***Acid and base induced degradation***

Under acidic conditions, degradation was dependent to the strength of hydrochloric acid and also exposure time (Table 5). No new peak was observed in the chromatogram after degradation (Figure 2b). The rate of degradation of clobazam tablet powder was comparatively higher than clobazam bulk powder under the same conditions (Table 6).

**Table 5:** The results of the stress degradation tests on clobazam bulk powder using different conditions

**Stress test condition**	**Solvent**	**Temperature**	**Time**	**% of clobazam**	**Peak homogeneity**
**Acidic**	1 M HCl	Room temperature	1 h	85.2	Pass
	2 M HCl	Room temperature	2 days	57.8	
	2 M HCl	60°C	1 h	71.4	
**Basic**	0.1 M NaOH	Room temperature	1 h	64.8	Pass
	0.1 M NaOH	Room temperature	48 h	1.2	
	0.1 M NaOH	60°C	1 h	13.8	
**Oxidative**	3% H_2_O_2_	Room temperature	48 h	75.3	Pass
	3% H_2_O_2_	60°C	1 h	91.4	
**Photolytic**					
UV light	Solid form	Room temperature	5 days	98.1	Pass
UV light	Water	Room temperature	5 days	65.0	
Visible light	Solid form	Room temperature	5 days	99.8	Pass
Visible light	Water	Room temperature	5 days	95.2	
**Heat**	Solid form	80°C	5 days	99.9	Pass
	Water	80°C	5 days	99.8	

**Table 6:** The results of the stress degradation tests on clobazam tablet powder using different conditions

**Stress test condition**	**Solvent**	**Temperature**	**Time**	**% of clobazam**	**Peak homogeneity**
**Acidic**	2 M HCl	60°C	1 h	74.4	Pass
**Basic**	0.1 M NaOH	Room temperature	1 h	42.3	Pass
**Oxidative**	3% H_2_O_2_	60°C	1 h	87.3	Pass
**Photolytic**					
UV light	Solid form	Room temperature	5 days	87.1	Pass
UV light	Water	Room temperature	5 days	24.9	
Visible light	Solid form	Room temperature	5 days	99.5	Pass
Visible light	Water	Room temperature	5 days	70.0	
**Heat**	Solid form	80°C	5 days	51.2	Pass
	Water	80°C	5 days	55.1	

In the presence of 0.1 M NaOH solution, a very fast degradation was observed. The degradation was about 35% and 99% after 1 h or 48 h at room temperature (Table 5). The degradation of tablet powder was more under the same conditions (Table 6). Under basic conditions, the formation of a small peak at the retention time of 1.2 min was evident (Figure 2c). Also a white crystal was produced which was increased by increasing the exposure time. After 48 h exposure time at room temperature, the complete degradation of clobazam to this product was observed.

#### ***Hydrogen peroxide induced degradation***

Significant degradation of clobazam bulk powder and also tablet powder was observed after exposure of clobazam to hydrogen peroxide at room temperature or 60°C (Tables 5 and 6). The hydrogen peroxide peak was observed at the retention time of about 1.2 without any other peak related to degradation products of clobazam (Figure 2d).

#### ***Photolytic degradation***

Clobazam bulk powder was relatively stable in solid state after exposure to visible or UV-light. On the other hand, the aqueous solution of clobazam bulk powder showed significant degradation of about 5% and 35% (Table 5). Clobazam tablet powder showed about 13% degradation after 5 days exposure to UV light (Table 6 and Figure 2e)). Higher degradation rates were observed for the aqueous solution of tablet powder under visible light (30%) or UV light (75%) after 5 Days (Table 6).

#### ***Heat degradation***

Clobazam bulk powder was relatively stable under heat after 5 days (Table 5). On the other hand, the clobazam tablet powder and its aqueous solution were significantly labile under heat and about 49% and 45% of degradation was observed after 5 days exposure to 80°C (Table 6 and Figure 2f).

#### ***Peak purity studies***

The peak purity of the samples resulted from stress degradation was checked by using a photo diode array detector. Peak purity results derived from the PDA detector confirmed that the clobazam peak was homogeneous and pure and there is no interfering peak from the diluents or degradation products. These results confirmed the stability indicating capability of the proposed method for determination of clobazam in the presence of its degradation products.

#### ***Characterization of the basic degradation product***

**Figure 3:**
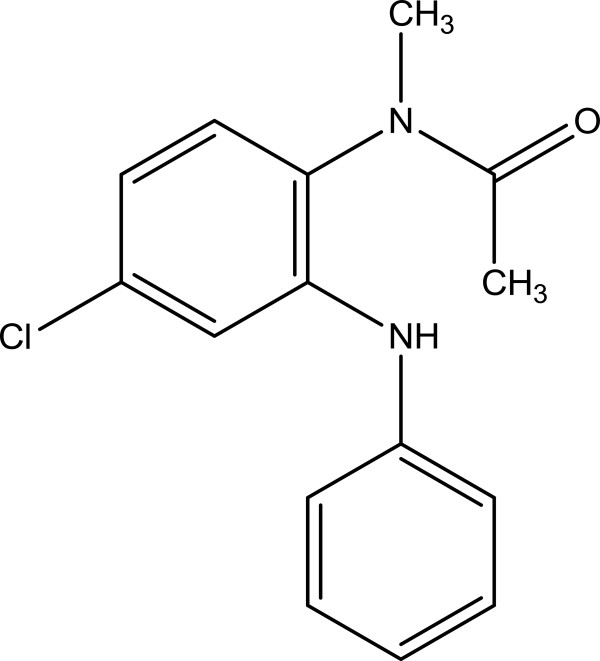
Chemical structure of degradation product of clobazam in basic medium.

**Figure 4:**
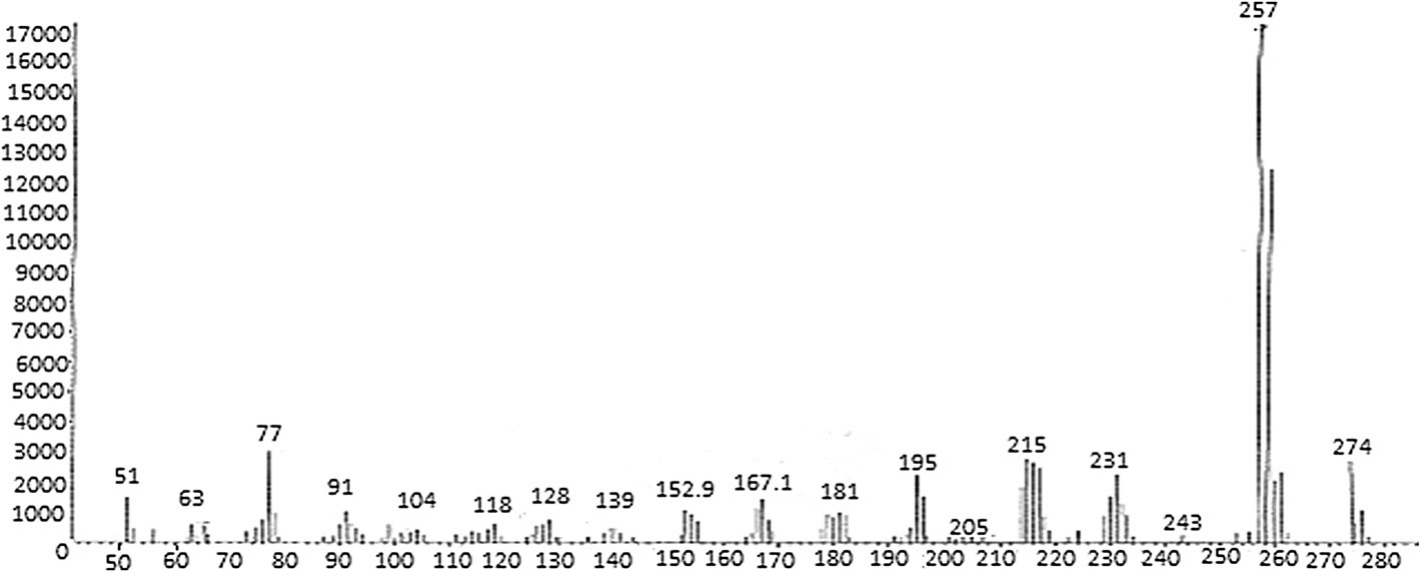
Mass spectrum of degradation product of clobazam in basic medium.

According to the experimental method, the degradation product in basic conditions was isolated. The isolated compound was pure and showed a single peak at the retention time of 7.5 (Figure 2g). On the basis of the ^1^H-NMR, mass and IR spectral data, the chemical structure of this product was assigned as N-[4-chloro-2-(phenyl amino) phenyl]-N-methylacetamide (Figure 3), impurity E, which has been reported in the European Pharmacopeia. The spectral data are as followed:

^1^H-NMR (CDCl_3_): δ (ppm); 1.93 (s, 3H, CH_3_-CO), 3.23 (s, 3H, NCH_3_), 6.84 (dd, J = 8.1 Hz and J = 2.0 Hz, 2H), 7.03 (d, J = 8.1 Hz, 1H), 7.10-7.17 (m, 3H), 7.25 (d, J = 2.0 Hz, 1H), 7.34-7.41 (m, 2H).Mass: m/z (%) 276 (7), 274 (21), 259 (33), 257 (100), 231 (15), 215 (18), 195 (13), 181 (7), 167 (8), 153 (7), 91 (7) (Figure 4).

IR (KBr): ν cm^−1^ 3267 (NH), 1644 (CO).

### Analysis of tablet dosage form

The active ingredient in clobazam tablets (10 mg) was determined using the developed method. Good agreement with the label claimed amount (10.29 ± 0.11 mg per tablet) was observed with no interference from excipeients.

## Conclusion

In this study clobazam was exposed to different stress conditions recommended in ICH guidelines. In the developed HPLC method, clobazam and its degradation products were resolved in a single isocratic run. Clobazam showed significant degradation under basic, acidic or oxidative conditions. The bulk drug remained unaffected under heat, UV light or visible light in the solid form, while significant degradation in these conditions was observed in aqueous solutions. The tablet powder was more labile under studied degradation conditions. The main degradation product under 0.1 M NaOH was separated and its chemical structure was elucidated. The proposed analytical method was relatively simple and accurate and could be used for the determination of clobazam in pharmaceutical dosage forms in the presence of its degradation products.

## Competing interests

The authors declare that there is no competing interests.

## Authors' contribution

ES has supervised the project and prepared the article. ADE has done the experimental part of the project. RA has performed the peak purity tests and obtained the mass spectrum. MA has performed the characterization of the degradation product. All authors read and approved the final manuscript.
